# Comprehensive assessment of fine motor movement and cognitive function among older adults in China: a cross-sectional study

**DOI:** 10.1186/s12877-024-04725-8

**Published:** 2024-01-31

**Authors:** Jie Zhang, Ye-Jing Zhao, Jun-Yi Wang, Han Cui, Shaojie Li, Xue Meng, Rui-Yu Cai, Juan Xie, Su-Ya Sun, Yao Yao, Jing Li

**Affiliations:** 1 Department of Geriatric Medicine, Beijing Hospital, National Center of Gerontology, Institute of Geriatric Medicine, Chinese Academy of Medical Sciences, Beijing, P. R. China; 2 Beijing Normal University, Beijing, China; 3 Office of the National Clinical Research Center for Geriatric Diseases, Beijing Hospital, Institute of Geriatric Medicine, National Center of Gerontology, Chinese Academy of Medical Sciences, Beijing, P. R. China; 4 Department of Geriatric Medicine, Zhucheng People’s Hospital, Weifang City, Shandong Province China; 5 Geriatric Department, Hefei First People’s Hospital, Hefei, China; 6 Department of Geriatrics, Tangshan Gong Ren Hospital, Tangshan, Hebei China; 7 China Center for Health Development Studies, Peking University, Beijing, China

**Keywords:** Fine motor movement, Cognitive function, MEMS motion capture technology, Various dimensions assessment

## Abstract

**Background:**

Fine motor skills are closely related to cognitive function. However, there is currently no comprehensive assessment of fine motor movement and how it corresponds with cognitive function. To conduct a complete assessment of fine motor and clarify the relationship between various dimensions of fine motor and cognitive function.

**Methods:**

We conducted a cross-sectional study with 267 community-based participants aged ≥ 60 years in Beijing, China. We assessed four tests performance and gathered detailed fine motor indicators using Micro-Electro-Mechanical System (MEMS) motion capture technology. The wearable MEMS device provided us with precise fine motion metrics, while Chinese version of the Montreal Cognitive Assessment (MoCA) was used to assess cognitive function. We adopted logistic regression to analyze the relationship between fine motor movement and cognitive function.

**Results:**

129 (48.3%) of the participants had cognitive impairment. The vast majority of fine motor movements have independent linear correlations with MoCA-BJ scores. According to logistic regression analysis, completion time in the Same-pattern tapping test (OR = 1.033, 95%CI = 1.003–1.063), Completion time of non-dominant hand in the Pieces flipping test (OR = 1.006, 95%CI = 1.000-1.011), and trajectory distance of dominant hand in the Pegboard test (OR = 1.044, 95%CI = 1.010–1.068), which represents dexterity, are related to cognitive impairment. Coordination, represented by lag time between hands in the Same-pattern tapping (OR = 1.663, 95%CI = 1.131–2.444), is correlated with cognitive impairment. Coverage in the Dual-hand drawing test as an important indicator of stability is negatively correlated with cognitive function (OR = 0.709, 95%CI = 0.6501–0.959). Based on the above 5-feature model showed consistently high accuracy and sensitivity at the MoCA-BJ score (ACU = 0.80–0.87).

**Conclusions:**

The results of a comprehensive fine-motor assessment that integrates dexterity, coordination, and stability are closely related to cognitive functioning. Fine motor movement has the potential to be a reliable predictor of cognitive impairment.

## Introduction

With a growing global aging population, cognitive impairment has been a public health issue that is the major cause of disability and dependency in older adults. The prevalence of cognitive impairment in older individuals is high, according to the World Health Organization, dementia had an estimated 55.2 million people worldwide in 2019, and it is predicted to reach 78 million by 2030 [1]. Dementia imposes a huge financial and caregiving burden; the global cost of dementia was estimated to be 1.3 trillion dollars in 2019 [1]. However, cognitive decline is a continuous and gradual accumulative process, there is a preclinical stage such as mild cognitive impairment (MCI) before progressing to dementia, which is the crucial stage in slowing the progression to dementia. A recent systematic review showed that the prevalence of MCI ranged from 1.2 to 87% [2]. Effective and accurate identification of MCI and intervention is essential for dementia prevention.

At present, the identification of early stages of cognitive decline is mostly dependent on the identification of biomarkers in structural magnetic resonance imaging, blood testing, and cerebrospinal fluid [3–6]. However, due to their drawbacks such as high cost and traumatic effects, it is difficult to implement these methods on a large scale. Therefore, there is a need to explore innovative, non-invasive, and relatively inexpensive approaches to identifying cognitive impairment.

Fine motor movements rely on small, precise movements of the hands and fingers that require brain coordination, and fine motor function is usually evaluated by dexterity, coordination, and stability [7, 8]. Numerous studies have found that deficits in cognition are associated with weak fine motor function [9–14]. For example, MCI and dementia patients have worse finger dexterity than normal older adults [9–11]. Meanwhile, bimanual coordination is also associated with cognitive functioning [12], going further, Torre’s and Roman-Liu’s research suggests that bimanual coordination exercises enhance cognitive functioning [13, 14].

However, previous studies have only considered a single aspect of fine motor [9–11], which makes it difficult to comprehensively and thoroughly explain the effects of fine motor and cognitive functions. There is a relative scarcity of studies based on comprehensive assessment of fine motor movement to explore the relationship with cognitive functioning. It is a fact that fine motor movements are complex control processes that require multi-dimensional involvement of hand and finger dexterity, coordination, stability, etc. It may be more appropriate to conduct a combined assessment of fine motor function and analyze its relationship with cognition. On the other hand, previous studies are based on western population or developed countries and lack validation in developing countries such as China, the country with the largest number of elderly people in the world. We are interested in developing a non-invasive predictive tool for early identification of cognitive impairment in developing countries. Therefore, in this study, Micro-Electro-Mechanical System (MEMS) was used to obtain fine-motor characteristics and to investigate the correlation between hand fine-motor and its main dimensions and cognitive functions.

## Methods

### Study design

We conducted a cross-sectional study conducted in Beijing, China, from 2022.12 to 2023.2. The study was approved by the Ethics Committee of Beijing Hospital (Approval no: 2021BJYYEC-291-01).

### Participants

Our study participants were recruited in the community through convenience sampling methods. The inclusion criteria included age ≥ 60 years, ability to complete cognitive and hand fine motor assessments, and voluntary participation with informed consent. We excluded those with The Beijing version of the Montreal Cognitive Assessment (MoCA-BJ) [15] ≤ 17; the existence of neurological injuries affecting fine motor function, such as stroke, Parkinson’s disease, brachial plexus injury, etc.; with skeletal muscle injuries that affect fine motor function, such as tendon injuries, hand trauma, joint dislocation, fractures, etc.; had a significant health event within the last 6 months including acute coronary events, severe infections, major surgery; and participating in other interventional clinical trials. 302 people were recruited and 287 were finally included in the study, excluding participants who did not meet the inclusion criteria.

### Cognitive function

We used the Beijing version of the Montreal Cognitive Assessment (MoCA-BJ) [15] to assess participants’ cognitive function, which is a screening tool widely used to assess cognitive function. The MoCA-BJ covers a range of cognitive domains, such as memory, language, attention, abstract thinking, orientation, visuospatial structural skills, and executive functioning, for a total score of 30. Cognitive impairment (CI) was defined as a MoCA-BJ score less than 26 [15, 16], and participants with MoCA-BJ ≥ 26 were considered cognitive healthy (CH). Internal consistency reliability by Cronbach’s alpha of MoCA-BJ was 0.73, and Intra-class correlation coefficient (ICC) was 0.87 [15].

### Fine motor movement assessment

#### Capture of fine motion movement

**Fig. 1:**
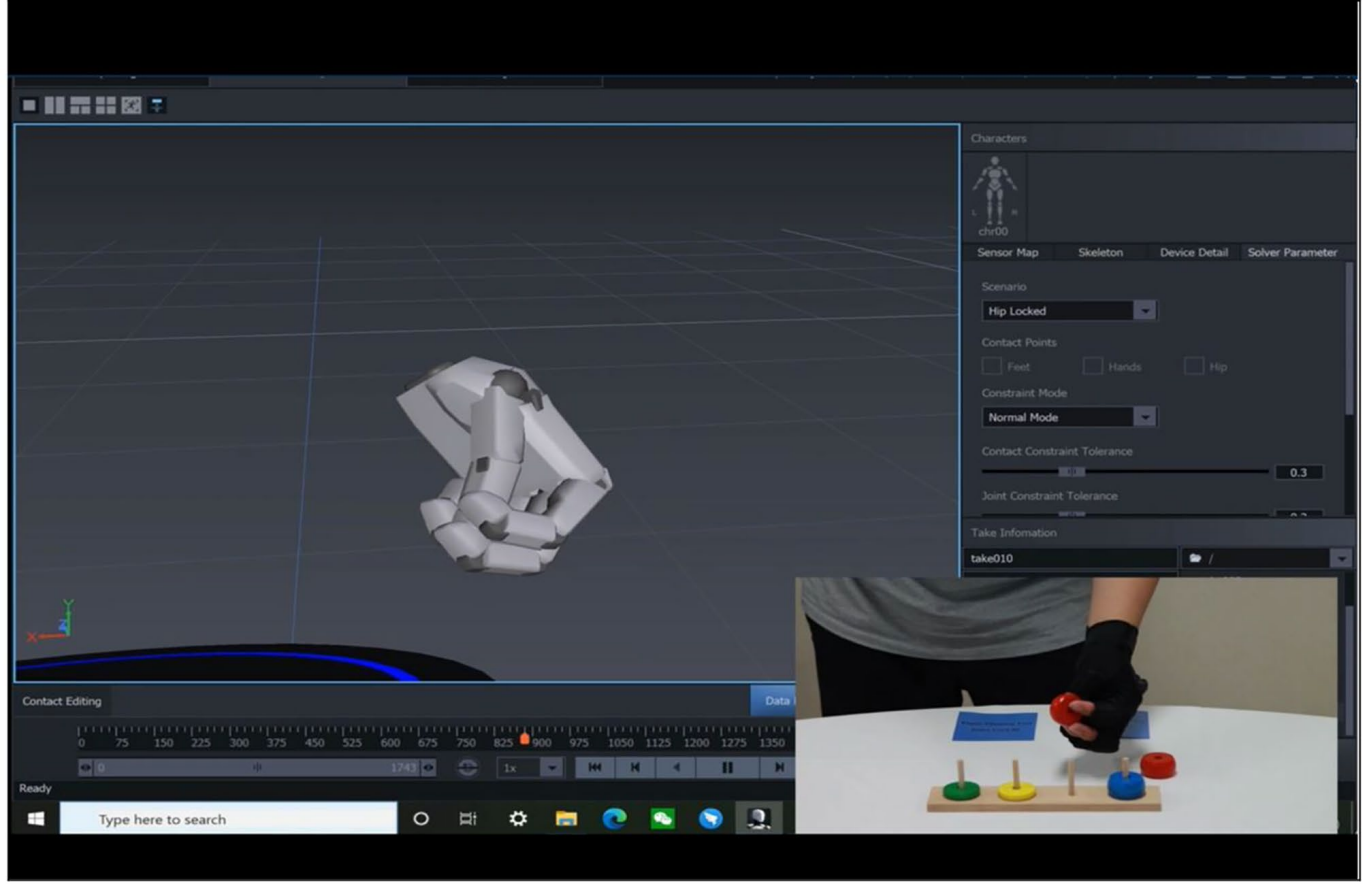
Fine motion movement capture by The perception Neuron® system

Fine motor movement were collected by The Perception Neuron® system (NOITOM, Beijing, China), which is a wearable device. The Perception Neuron® system has been applied within virtual reality interaction, medical diagnosis, and rehabilitation robot control [17–19]. This system is based on Micro-Electro-Mechanical System (MEMS) inertial motion capture technology which includes a MEMS inertial sensor, data forwarding router, and computer terminal. The tests were presented in a virtualized form in the monitor, participants completed test-oriented executions with wearable MEMS inertial motion sensors. A single hand is equipped with 6 inertial sensors (five for the second knuckle of each finger and one for the back of the hand) to collect the motor data of sensors in various parts, and the data is forwarded to the computer terminal through the router in the form of wireless forwarding. To prevent interference in the same frequency band, Time Division Multiple Assessment (TDMA) is added to a data transmission. Through model rendering, the real-time 3D model and motion data of hand joint movement were rendered (Fig. 1). Participants were evaluated by trained and established physicians. The fine motor assessment process and notes were explained to the participants before the assessment, and 10 min were provided for the participants to familiarize the assessment system. Once the test was started, participants were given only 1 opportunity to complete the test. The duration of the fine motor assessment was approximately 15 min. The computer system standardizes and saves the parameters obtained.

#### Fine motor movement assessment tests

**Fig. 2:**
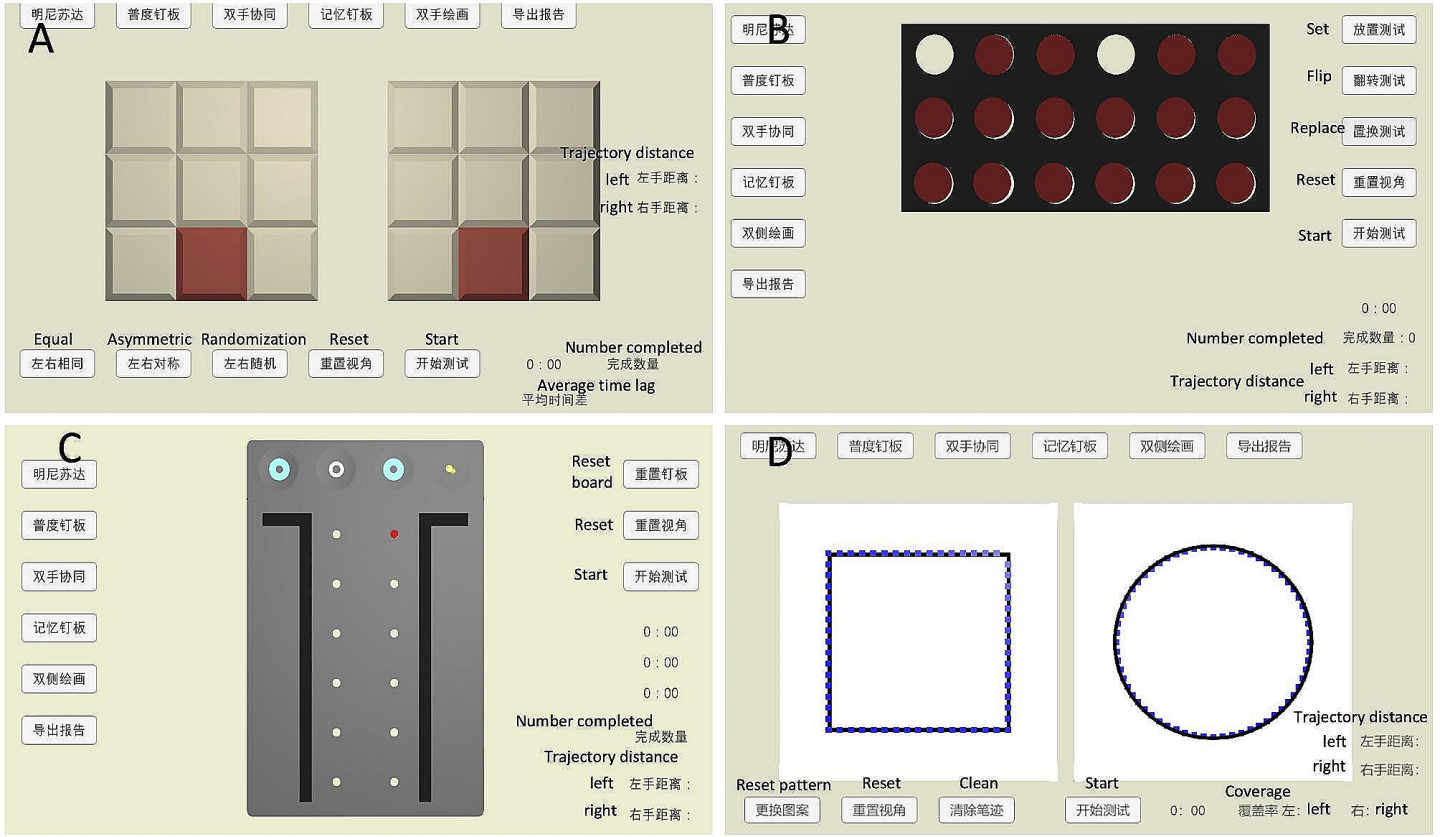
Fine motor movement assessment test. (**A**) Same-pattern tapping test. (**B**) Pieces flipping test. (**C**) Pegboard test. (**D**) Dual-hand drawing test

Four tests were created to evaluate performance of fine motor movement, and participants were required to complete those tests sequentially (Fig. 2).

#### Same-pattern tapping test

Two identical nine-cell squares are displayed on the screen, one for the left hand and the other for the right hand. When the test started, color cues appeared at different cells of the squares. The participants were instructed to touch the color-lit squares as quickly as possible, and both hands must be touched simultaneously. Completion time and time difference between hands were captured and recorded.

#### Pieces flipping test

There are 18 pieces with red fronts and white backs placed on a blackboard. Participants take the pieces out, flip them over, and return them to their original positions as quickly as they can with two separate hands. The completion time of both hands was recorded by the assessment system.

#### Pegboard test

The Pegboard is a common test used to assess hand dexterity [8], this study modified the traditional the Pegboard test. Participants were instructed to pick up 16 nails at the top of the screen with their thumb, index, and middle fingers and place them sequentially in two columns of nail holes. The test required rhythmic completion of the left and right hands at top speed. The completion time and trajectory distance of both hands were recorded.

#### Dual-hand drawing test

A plot of a circle and a square appeared on the screen, participants followed the trajectory cues to depict them simultaneously, which entails a circle in the left hand and a square in the right hand, the timing was completed once all graphics were completed. We captured and recorded the completion time and coverage of hands.

### Confounding variable

We chose covariates variable based on previous studies [5, 20, 21]. Confounders were selected if they were considered to be correlated with both fine motor function and cognition and not as intermediaries for association between them. We included demographic features (age, sex, marital status, education, living alone or not, family income, and previous occupation type), health status and lifestyle (smoking, drinking alcohol, BMI, and grip strength), and chronic diseases, including diabetes, hypertension, Chronic Obstructive Pulmonary Disease (COPD), Atrial fibrillation (AF), Chronic heart failure (CHF), Coronary heart disease (CHD).

### Statistical analyses

The data were analyzed using SPSS Statistics Version 25.0 for Windows. Continuous variables conforming to normal distribution are described as mean ± SD. IQRs are used to describe continuous variables for nonnormally distributed data. Count data are described by frequency. The continuous variables were compared using unpaired Student’s t-tests for normal distribution or Wilcoxon-Mann-Whitney tests for nonnormal distributions. The categorical variables were compared using χ2 tests.

Firstly, we used nonparametric tests to compare fine motor parameters between the HC and CI groups. Then, we performed univariate and multivariate linear regression to the analyze relationship between fine motor indexes and MoCA-BJ score. Meanwhile, we adopted logistic regression to explain the relationship between fine motor skills and cognitive impairment, the odds ratios (ORs) and 95% confidence intervals (CIs) were used to test the significance of the differences between the two groups, and *p* ≤ 0.05 was considered statistically significant in all analyses.

After identifying the five fine motor test predictors of cognitive decline via regression analysis, we aim to further assess their diagnostic performance to classify various levels of cognitive decline. To this end, we employed ROC curves at various MoCA-BJ score cutoff (23–28) and calculated the area under curve (AUC). More specifically, we first evaluated whether participant performance of all previously identified fine motor tests is more sensitive and accurate to detect minimal cognitive decline (i.e., classifying participants with MoCA-BJ score < 28 vs. the rest), or a higher degree (classifying participants with MoCA-BJ score < 23 vs. the rest), and all levels in between. Furthermore, since we aim to develop fine motor test-based metrics to detect early cognitive decline, we try to improve the diagnostic performance to better classify participants with mild MoCA-BJ score decline (i.e., MoCA-BJ score < 28 vs. the rest). To this end, we employed recursive feature elimination (RFE) on the existing 5 features to select a subset of 3 fine motor tests that showed higher sensitivity and accuracy for identifying participants with only a mild decrease in MoCA-BJ score. The scikit-learn v1.3.0 package was used for model construction and testing under Python interpreter 3.11.1. Models based on a logistic regression classifier were trained using 70% randomly selected participants with the default hyperparameters, where the rest were used for evaluation purposes and calculating the AUCs. We first selected all fine motor tests found to be significantly correlated with the incidence of MCI in the adjusted logistic regression for model training. Figures were generated using the matplotlib package v3.8.0rc1.

## Results

### Participants characteristics

The characteristics of the subjects are presented in Table 1. The age of the study subjects ranged from 60 to 82 years and the mean age was 66.42 ± 4.76 years. Most participants (68.2%) were females. The MoCA-BJ score was 25.00 ± 3.49, and 48.3% were considered to have cognitive impairment. Participants in the CI group were older, with fewer high-income families, mental workers, unmarried, widowed, and divorced enrollers. The cognitive impairment group had more diabetics and higher BMI levels than the CH group. There was a difference in education level between the two groups (Table 1).

**Table 1:** Characteristics of the subjects

	ALL (*n* = 267)	CH (*n* = 138)	CI (*n* = 129)	*P* value
Age, y	66.42 ± 4.76	65.39 ± 4.66	67.53 ± 4.64	**< 0.001**
Female, *n* (%)	182 (68.2)	99(54.4)	83(45.6)	0.195
Marital Statue (Unmarried, widowed, and divorced), *n* (%)	19(7.1)	14(10.1)	5(3.9)	**0.046**
Living alone, *n* (%)	11(4.1)	8(5.8)	3(2.3)	0.154
Education, *n* (%)				**< 0.001**
Lower	35(13.1)	9(6.5)	26(20.2)	
Secondary	29(29.6)	35(25.4)	44(34.1)	
High	153(57.3)	94(68.1)	59(45.7)	
Previous occupation type (Mental labor), *n* (%)	155(58.1)	94(68.1)	61(47.3)	**0.001**
High family income, *n* (%)	183(68.5)	106(76.8)	77(59.7)	**0.003**
Smoking, *n* (%)	32(12.0)	13(9.4)	19(14.7)	0.182
Drinking alcohol, *n* (%)	27(10.1)	13(9.4)	14(10.9)	0.698
BMI, kg/m^2^	24.15 ± 2.99	23.80 ± 2.86	24.54 ± 3.08	**0.044**
Grip strength, kg				
Dominant hand	29.24 ± 9.42	29.19 ± 8.51	29.30 ± 10.35	0.928
Non-dominant hand	27.26 ± 8.99	27.62 ± 8.11	26.87 ± 9.87	0.500
Diabetes, *n* (%)	45(16.9)	17(12.3)	28(21.7)	**0.041**
Hypertension, *n* (%)	112(41.9)	55(39.9)	57(44.2)	0.474
COPD, *n* (%)	2(0.7)	1(0.7)	1(0.8)	0.962
AF, *n* (%)	6(2.2)	2(1.4)	4(3.1)	0.363
CHF, *n* (%)	2(0.7)	1(0.7)	1(0.8)	0.962
CHD, *n* (%)	5(1.9)	1(0.7)	4(3.1)	0.152

Education: Lower: middle school or below, Secondary: high school, High: college or above

High family income: ≥10 000RMB/m

BMI, body mass index; COPD, chronic obstructive pulmonary disease; AF, atrial fibrillation; CHF, chronic heart failure; CHD, coronary heart disease

### Performance of fine motor in CH group and CI group

In general, the CH group took less time to finish the majority of testing tests. Participants in the CI group had more time difference between hands for the Same-Pattern Tapping Test. In the Pegboard test, the mobility distance in the CH group is shorter, and participants in the CH group performed better in coverage of the Dual-hand drawing test (Table 2).

**Table 2:** Comparisons of differences in fine motor function between CH group and CI group

	CH (*n* = 138)	CI (*n* = 129)	*P* value
Same-pattern tapping test			
Completion time (s)	22.88(19.78, 28.23)	25.76(21.39, 34.28)	**0.001**
Time difference between hands (s)	0.33(0.18, 0.58)	0.60(0.23, 1.10)	**< 0.001**
Pieces flipping test			
Completion time_ dominant hand (s)	81.95(58.79, 114.49)	93.89(68.33, 128.79)	0.079
Completion time_ non-dominant hand (s)	84.79(61.97, 109.36)	102.61(75.27, 138.30)	**< 0.001**
Pegboard test			
Completion time (s)	115.35(87.58, 166.34)	137.13(102.15, 220.59)	**0.003**
Trajectory distance_ dominant hand (cm)	15.17(10.49, 20.75)	19.19(14.63, 26.59)	**< 0.001**
Trajectory distance_ non-dominant hand (cm)	15.42(11.03, 21.10)	18.55(13.00, 25.61)	**0.001**
Dual-hand drawing test			
Completion time (s)	22.22(17.40, 27.22)	21.54(15.54, 32.79)	0.866
Coverage_ dominant hand (%/s)	3.19(2.39, 3.89)	2.55(1.70, 3.64)	**< 0.001**
Coverage_ non-dominant hand (%/s)	2.75(2.16, 3.76)	2.43(1.58, 3.37)	**0.009**
Coverage difference between hands (%/s)	0.70(0.29, 1.13)	0.65(0.33, 1.26)	0.563

### Correlation between the fine motor and cognitive function

As shown in Table 3, the Completion time of the Same-pattern tapping test, the Pieces flipping test, the Pegboard test, and the time lag between hands in the Same-pattern tapping test were negatively correlated with the MoCA-BJ score (*β*=-0.217 ~ -0.119, *P* < 0.05). Meanwhile, the trajectory distance of hands in the Pegboard test was negatively correlated with the MoCA-BJ score (*β*=-0.231 ~ -0.143, *P* < 0.05). Coverage of the dominant hand and non-dominant hand in the Dual-hand drawing test were all positively correlated with the MoCA-BJ (*β* = 0. 179 ~ 0.213, *P* < 0.05). Whereas, there was no correlation between completion time and coverage in the Dual-hand drawing test with MoCA.

**Table 3:** Univariate and multivariate linear regression analysis of the fine motor and MoCA

	unadjusted model		adjusted model ^a^	
	β Coefficients (95% Confidence Interval)	*p*-Value	β Coefficients (95% Confidence Interval)	*p*-Value
Same-pattern tapping test				
Completion time (s)	-0.217 (-0.081, -0.024)	**< 0.001**	-0.158 (-0.067, -0.010)	**0.009**
Time difference between hands (s)	-0.265 (-1.361, -0.528)	**< 0.001**	-0.204 (-1.147, -0.309)	**0.001**
Pieces flipping test				
Completion time_ dominant hand (s)	-0.143(-0.019, -0.002)	**0.019**	-0.066(-0.013,0.004)	0.277
Completion time_ non-dominant hand (s)	-0.231(-0.023, -0.008)	**< 0.001**	-0.176(-0.019, -0.004)	**0.003**
Pegboard test				
Completion time (s)	-0.199 (-0.013, -0.003)	**0.001**	-0.132(-0.010, -0.001)	**0.027**
Trajectory distance_ dominant hand (cm)	-0.246 (-0.147, -0.052)	**< 0.001**	-0.169(-0.115, -0.021)	**0.004**
Trajectory distance_ non-dominant hand (cm)	-0.202 (-0.135, -0.035)	**0.001**	-0.129(-0.103, -0.006)	**0.029**
Dual-hand drawing test				
Completion time (s)	-0.018 (-0.046, 0.034)	0.768	-0.011(-0.042, 0.035)	0.846
Coverage_ dominant hand (%/s)	0.213 (0.219, 0.764)	**< 0.001**	0.183(0.155, 0.689)	**0.002**
Coverage_ non-dominant hand (%/s)	0.179 (0.153, 0.761)	**0.003**	0.153(0.094, 0.683)	**0.010**
Coverage difference between hands (%/s)	-0.064 (-0.739, 0.228)	0.299	0.065(-0.729, 0.208)	0.274

^a^ adjusted for age, gender, marital status, education, previous occupation type, income, BMI, smoking, drinking alcohol, hypertension, and diabetes

In multivariate linear regression, we adjusted for covariates that differed in Table 2. In addition, gender, smoking, drinking alcohol and hypertension are considered important impact factors in cognitive impairment according to previous studies [20, 21], we also adjusted them in multivariate analysis. We found that the results were consistent with the results of the univariate analysis except for the completion time of the dominant hand in the Pieces flipping test.

We further analyzed the association between fine motor and cognitive impairment. We found that the Completion time and the time difference between hands in Same-pattern tapping test, Completion time of non-dominant hand in the Pieces flipping test, and trajectory distance of dominant hand in the Pegboard test were inversely associated with cognitive impairment (OR = 1.006 ~ 1.663, *P* < 0.05), and Coverage of dominant hand in Dual-hand drawing test were significant negatively associated with cognitive impairment (OR = 0.790, *P* < 0.05) (Table 4).

**Table 4:** Logistics regression analysis of the fine motor and cognitive impairment

	unadjusted model		adjusted model ^a^	
	OR (95% Confidence Interval)	*p*-Value	OR (95% Confidence Interval)	*p*-Value
Same-pattern tapping test				
Completion time (s)	1.039 (1.013, 1.066)	**0.003**	1.033 (1.003, 1.063)	**0.030**
Time difference between hands (s)	1.869 (1.287, 2.715)	**0.001**	1.663 (1.131, 2.444)	**0.010**
Pieces flipping test				
Completion time_ dominant hand (s)	1.006(1.000, 1.011)	**0.032**	1.002(0.996, 1.008)	0.517
Completion time_ non-dominant hand (s)	1.008(1.003, 1.014)	**0.002**	1.006(1.000, 1.011)	**0.040**
Pegboard test				
Completion time (s)	1.004 (1.001, 1.007)	**0.019**	1.003 (0.999, 1.006)	0.122
Trajectory distance_ dominant hand (cm)	1.060 (1.028, 1.092)	**< 0.001**	1.044 (1.010, 1.078)	**0.010**
Trajectory distance_ non-dominant hand (cm)	1.048 (1.017, 1.081)	**0.003**	1.033 (0.999, 1.068)	0.055
Dual-hand drawing test				
Completion time (s)	1.012 (0.989, 1.035)	0.325	1.012 (0.986, 1.039)	0.375
Coverage_ dominant hand (%/s)	0.769 (0.643, 0.921)	**0.004**	0.790 (0.650, 0.959)	**0.017**
Coverage_ non-dominant hand (%/s)	0.844 (0.702, 1.013)	0.069	0.856 (0.700, 1.046)	0.129
Coverage difference between hands (%/s)	1.175 (0.890, 1.552)	0.254	1.165 (0.843, 1.609)	0.356

^a^ adjusted for age, gender, marital status, education, previous occupation type, income, BMI, smoking, drinking alcohol, hypertension and diabetes

**Fig. 3:**
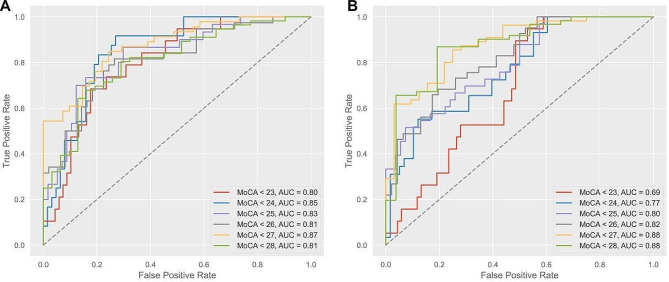
The receiver operating curves for fine motor predictors for cognitive impairment

Figure 3. Receiver operating characteristic (ROC) curves based on logistic regression model using (A) all five identified fine motor test predictors for cognitive impairment and (B) selected top three top features using recursive feature elimination (RFE). Combined diagnostic abilities of the 5-feauture model (A) reveals consistently high accuracy and sensitivity across various MoCA-BJ score cutoffs, while the 3-feature model (B) has superior performance for mild cognitive decline before the incidence of MCI.

## Discussion

We performed a comprehensive evaluation of fine motor function by MEMS and found that fine motor function was correlated with cognitive function, in addition to this, fine motor performance on a number of tests was independently correlated with cognitive impairment in older adults.

Our study found that 48.3% of old adults suffer from cognitive impairment, similar to other researchers’ findings on the elderly in China [22, 23], and consistent with previous consensus, we also found cognitive function to be correlated with age, education, income, and occupation type [1, 15]. Fine motor function of the hand is an important physiological function in the elderly. Our study has found a correlation between fine motor and cognitive function and cognitive decline in older adults.

Dexterity is one of the most important characteristics of fine motor. In our study, most of the fine motor function reflecting dexterity, including completion time in the Same-pattern tapping test and the Pieces flipping test, completion time, and trajectory distance in the Pegboard test, were negatively correlated with cognitive functioning. This result is consistent with previous studies which also revealed that finger dexterity was related to MMSE [24, 25]. We found that some dexterity indicators are still linked to cognitive impairment in a further logistic regression based on whether or not cognitive impairment is present. The same conclusion has been reached by previous studies on fine motor performance in MCI, dementia, and normal cognitive older adults [26, 27]. Potential mechanisms related to alterations in the cerebral cortex. It is widely known that the motor including fine motor is dominated by the cerebral cortex, Pre-motor and motor cortex are higher cortical centers that initiate movement. During aging, cortical atrophy affects cognitive function as well as finger dexterity. A recent study published in the Lancet demonstrates that older adults with intact cognitive function have better finger dexterity and slower atrophy rates of gray matter [28], which also illustrates the correlation between changes in the cerebral cortex and finger dexterity. However, logistics regression analysis suggests that only completion time on the non-dominant hand is associated with cognitive impairment in the Pieces Flip test after adjusting for confounding factors. This may be because the non-dominant hand is more difficult to control when executing an action, and control is more susceptible to cognitive functioning.

The coordination of hands decreases with aging, previous studies have found that older adults often struggle with bimanual coordination [3, 12, 29]. Indicators such as the lag time between hands during Same-pattern tapping and the coverage difference between hands in the Dual-hand drawing test demonstrate how well the hands synchronize when performing a test. After adjusting for covariates, the time difference between hands in the Same-pattern tapping test was shown to be independently correlated with cognition in both linear and logistic regressions. Some researchers have suggested that coordination may be related to the subcortical motor system [30]. The subcortical motor system is an important mediator of motor practice and sensorimotor integration, and is associated with both motor control and cognitive functions [31–32]. On the other hand, bimanual coordination is a complex test relying on multiple functional networks throughout the entire brain, previous studies suggest that impaired bimanual coordination in older adults with MCI and dementia is attributed to reduced neural network connectivity in the brain [33, 34]. however, our study did not find any association between cognitive functioning and the coverage difference between hands on a dual-hand drawing test, which is another indicator of bimanual coordination.

As an important feature of fine motor movement, stability is tightly linked to the ADL of older adults. The smoothness of the writing or drawing trajectory reflects the stability of the hands and finger control. In this study, to assess stability, we computed the coverage of smooth lines in a drawing test and made adjustments to the completion time. In our research, we discovered a negative correlation between the amount of two-handed coverage and MoCA-BJ scores. This finding aligns with previous studies [35, 36], Dahdal executed the Motor Performance Series (MLS) to analyze fine hand movements and found that Parkinson’s patients with MCI had poorer dynamic stability compared to those without MCI [37]. An important feature of human fine motor is the minimal jerk, which represents a swing and pause throughout a continuous movement [17], cognitive functions play a crucial role in the control of the jerk. In logistics regression, however, our study discovered a correlation between cognitive impairment and the stability of the dominant hand. This could be attributed to the fact that our study focused on dynamic stability. Some researchers have argued that the non-dominant hand typically plays a more stable role in daily activities, while the dominant hand takes on a more dynamic role [9]. As a result, the indicator of the dominant hand may be more sensitive in detecting cognitive impairment.

There are some limitations to be considered. Firstly, this is a cross-sectional study that only reveals the correlation between fine motor and cognitive functioning, but lacks a cohort study to explain the role of effects on cognitive development. Secondly, the present study only analyzed the relationship between fine motor and overall MoCA and lacked in-depth exploration of specific cognitive dimensions. Further studies target specific cognitive dimensions for feature collection and analyze their relationship with fine motor. Lastly, assisting with the preclinical diagnosis of dementia, such as MCI is even more valuable, however, the diagnosis of MCI is a complex process that requires a combination of daily life performance, imaging techniques, biomarkers, and psychometric assessments. While the present study evaluated cognitive functioning only through psychological scales, which is why MCI was not used in this study and cognitive impairment was instead applied to define older adults. Further, we will explore the relationship between fine motor and dementia in preclinical patients using standardized diagnostics.

## Conclusions

In summary, our study assessed various dimensions of fine motor function through a comparatively complete test system, confirming that there is a link between fine motor and cognitive. Additionally, it has been found that certain aspects of fine motor skills can be connected to cognitive impairment. The research findings provide a foundation for the further development of cognitively relevant fine motor assessment systems and also provide new ideas and perspectives for the early identification of cognitive impairment in the future.

## Data Availability

No datasets were generated or analysed during the current study.
